# Mitigating UV‑C Degradation in Polypropylene Using Hybrid TiO_2_/Few-Layer Graphene/Photostabilizer Systems

**DOI:** 10.1021/acsomega.5c08936

**Published:** 2025-10-27

**Authors:** Jessica C. Ferreira Gimenez, Robert Paiva, Sophia H. F. Bonatti, Lucas H. Staffa, Edenir Rodrigues Pereira-Filho, Emna Helal, Nicole R. Demarquette, Manoel G. P. Homem, Sandra A. Cruz

**Affiliations:** † Department of Chemistry, Exact Sciences and Technology Center (CCET), 67828 Federal University of São Carlos (UFSCar), Rodovia Washington Luís, Km 235, SP-310, São Carlos, São Paulo 13565-905, Brazil; ‡ Department of Mechanical Engineering, 14849 École de Technologie Supérieure (ÉTS), 1100 Notre-Dame St W, Montréal, Quebec H3C 1K3, Canada; § Department of Materials Engineering, Exact Sciences and Technology Center (CCET), Federal University of São Carlos (UFSCar), Rodovia Washington Luís, Km 235, SP-310, São Carlos, São Paulo 13565-905, Brazil; ∥ NanoXplore Inc., 4500 Thimens Blvd, Saint-Laurent, Montréal, Quebec H4R 2P2, Canada

## Abstract

Polypropylene (PP) is a versatile thermoplastic widely used in industrial fields. In medical devices, PP is preferred for applications that involve storing or coming into contact with biological fluids. However, when exposed to UV-C, commonly used as a cleansing tool in hospitals, PP undergoes a photodegradation process, resulting in chain scissions and branching reactions that impact the material properties and lifespan. Different photostabilizers can be used to enhance polymer resistance against UV, such as UV screeners like titanium dioxide rutile (TiO_2_), radical scavengers like Irganox B215, a commercial H-donor and peroxide scavenger, and, more recently, graphene and its derivatives like few-layer graphene (FLG). Graphene has gained attention as an alternative photostabilizer in polymers for having different types of UV photoprotection mechanisms, such as UV absorbers/screeners and radical scavengers. In this context, this study aimed to evaluate the effectiveness of FLG and Irganox B215 as radical scavengers in combination with TiO_2_ to minimize the effect of parallel radical formation (ROS) from TiO_2_ electron–hole reactions and from the PP photodegradation autocatalytic cycle. A Design of Experiments (DoE) approach was employed to identify the optimal UV-C photostabilization mixture. Infrared spectroscopy and rheological measurements were used to assess the effects of UV-C photodegradation on PP. Scanning electron microscopy and energy-dispersive X-ray spectroscopy were used to analyze the stabilizer distribution and dispersion, and electron paramagnetic resonance evaluate the effectiveness of FLG and Irganox B215 as radical scavengers. EPR results showed that mixing radical scavengers with TiO_2_ reduced OH formation by ∼30% for the FLG and ∼25% for the B215 mixture. Although the stabilizers exhibited poor dispersion but good distribution, the addition of FLG had a synergistic effect with TiO_2_. At the highest level (+1), i.e., TiO_2_ 3% and FLG 2% m/m, PP UV–C photoprotection was enhanced by diminishing chain scission and scavenging ROS from TiO_2_.

## Graphical abstract

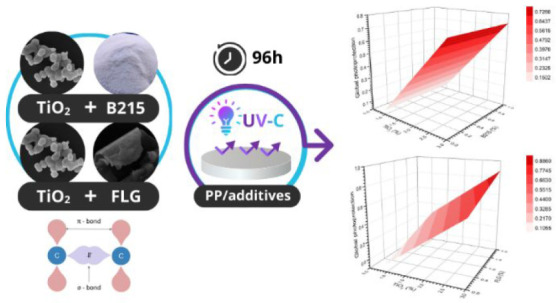

## 1 Introduction

Polypropylene (PP) is a versatile thermoplastic widely used across several industrial fields, from food packaging and automotive components to laboratory equipment and medical devices.^1^ Its biological inertia^2^ makes it a preferred material in medicine, especially for devices that store or come into contact with biological fluids.^1–3^ However, despite its numerous applications, PP faces challenges when exposed to UV–C light, commonly used in hospitals.^4^ This exposure initiates a photodegradation process, resulting in chain scissions and branching reactions that significantly impact the polymer’s molar mass and distribution, ultimately leading to a marked reduction in its physical and chemical properties and lifespan.

Numerous additives have been studied and used for their effectiveness in UV-A and UV-B photoprotection. These materials function at various stages of the photodegradation cycle as UV absorbers, screeners, quenchers, and radical scavengers.^5,6^ Among them, inorganic stabilizers, such as zinc oxide (ZnO)^7^ and titanium dioxide (TiO_2_), have been widely incorporated either in coatings^8,9^ or in composites,^10,11^ where they act as UV absorbers or screeners to shield polymers from harmful UV radiation. In addition, commercial organic antioxidants^12^ and, more recently, graphene and its derivatives^13–19^ have also shown significant potential in enhancing polymer UV resistance.

TiO_2_ exists in three crystalline phases: anatase, rutile, and brookite, each with distinct photoactivity and band gap values. The photocatalytic mechanism of single TiO_2_ in the presence of water and UV light is known in the literature.^20–23^ Although this compound acts as a photostabilizer, it generates reactive oxygen species (ROS), such as peroxides, oxygen in the singlet state, and hydroxyl radicals,^22^ which may induce further degradation in materials like polymers.^24–27^

Among the crystalline phases of TiO_2_, rutile presents relatively low photocatalytic activity and is widely used as a UV screener in polymer materials. Despite its low bandgap of 3.03 eV,^23^ rutile can still generate free radicals upon exposure to UV light, oxygen, and water. These radicals can attack the polymer and initiate further degradation reactions, ultimately reducing the polymer’s lifespan. Although some studies have explored the use of TiO_2_ for UV–C stabilization,^8,10,11,24^ none have fully addressed the parallel formation of radicals resulting from TiO_2_’s electron–hole reactions.

To mitigate the formation of ROS by TiO_2_, the addition of radical scavengers appears to be a promising solution. One well-known antioxidant in this context is Irganox B215, a blend of Irganox 1010, a phenolic antioxidant that acts as an H-donor, and Irgafos 168, an organophosphate that decomposes peroxide radicals.^12^ This commercial blend has shown efficacy as a photostabilizer in polymers, although it is widely used as a stabilizer for processing.

Recently, graphene and its derivatives gained attention as alternative photostabilizers in polymers.^13–19^ With a unique two-dimensional hexagonal honeycomb structure composed of a single layer of sp^2^ carbon atoms, graphene possesses diverse and interesting properties that make it suitable for various applications in polymer composites.^28,29^ As a photostabilizer, graphene and its derivatives can function as UV absorbers/screeners, physical barriers, and radical scavengers.^6^

Although monolayer graphene holds potential, its high synthesis cost and complexity make it challenging to scale up for industrial applications. Instead, few-layer graphene (FLG), a commercially available form typically consisting of 5 to 10 layers of sp^2^ carbon atoms, offers a more cost-effective and practical alternative. FLG is produced through the mechanochemical exfoliation of graphite, a process that the industry has readily adopted^6^ and has shown promising results.

Several studies have demonstrated the potential of graphene for UV-A and UV-B photoprotection.^13–19^ In the case of FLG, a recent study on high-density polyethylene (HDPE)/FLG composites showed that commercial-grade graphene effectively acts as a UV screener and radical scavenger against UV-A radiation.^18^ For the UV-C range, only one study has demonstrated the use of graphene oxide as a photostabilizer,^30^ and no studies have explored the use of FLG for photostabilization in this UV range. Furthermore, the potential of combining graphene or other radical scavengers with TiO_2_ to enhance polymer photostabilization and scavenge ROS from the TiO_2_ parallel electron–hole reaction remains largely unexplored.

In this context, the present study aimed to evaluate the effectiveness of FLG and Irganox B215 as radical scavengers, not only in protecting polymers against UV–C-induced photodegradation but also in combination with TiO_2_ to minimize the effect of parallel radical formation due to electron–hole reactions. To identify the optimal UV-C photostabilization mixture, the Design of Experiments (DoE) approach was employed. The photodegradation effects in the polymer composites were assessed using Infrared Spectroscopy (FTIR-ATR) and rheological analyses. Stabilizer distribution and dispersion were analyzed by using scanning electron microscopy and energy dispersive X-ray spectroscopy (SEM-EDS). Additionally, electron paramagnetic resonance (EPR) experiments were conducted to evaluate the effectiveness of FLG and Irganox B215 as radical scavengers when combined with TiO_2_.

## 2 Materials and Methods

### 2.1 Materials

The homopolymer polypropylene pellets used in this study were of HP 523J grade, supplied by BRASKEM, Brazil, with a melt flow index (MFI) of 3.1 g/10 min (ASTM 1238, 230 °C, 2.16 kg) and a density of 0.902 g/cm^3^ (ASTM D792). Titanium dioxide in the rutile phase, with a purity of >99% and an average particle size of 100 nm, was provided by MERCK, Brazil. Irganox B215, a blend of Irgafos 168 and Irganox 1010 in a 2:1 ratio, was supplied by BASF, Brazil. Few-layer graphene Black 3X, with 6 to 10 layers and a bulk density of 0.18 g/cm^3^, was provided by NanoXplore, Canada. More detailed characterization of TiO_2_ and FLG can be found in the Supporting Information, Figures S1, S2, and S3.

### 2.2 Composite Preparation

Polypropylene (PP) composites with TiO_2_ + FLG and TiO_2_ + Irganox B215 were prepared in a Thermo Fisher Process 11 Parallel Twin-Screw Extruder. The extrusion process was carried out at a constant barrel temperature of 200 °C and a screw speed of 100 rpm. The most appropriate mass percentages (%m/m) of TiO_2_ + FLG and TiO_2_ + Irganox B215, required for optimal UV-C photoprotection, were determined through a Design of Experiment (DOE) approach, specifically a 2^2^ modified factorial planning design with a central point.

For photodegradation exposure, samples with thicknesses ranging from 0.1 to 0.2 mm were produced by hot pressing in a TIL MARCON MPH-10 hydraulic press at 200 °C under a pressure of 0.5 tons.

### 2.3 UV-C Photodegradation

The samples were exposed to UV-C light in a metallic chamber with a 33 cm diameter and a 29 cm height, equipped with a fan on one side to ensure complete ozone removal. The UV-C source consisted of two commercial Hg lamps (Philips TUV 4 W) emitting at a peak wavelength of 254 nm (471 kJ/mol), positioned 26 cm apart. Each sample positioned 10 cm from the lamps was exposed on both sides for 96 h. The irradiation intensity on the sample surface was approximately 1.3 mW/cm^2^. Following the exposure, the samples were immediately analyzed by using rheological measurements and Fourier-transform infrared spectroscopy with an attenuated total reflectance (FTIR-ATR) accessory.

### 2.4 Physicochemical Analysis

Infrared spectra of the exposed samples were used as one of the responses in the DoE. These spectra were obtained by using a Thermo Scientific Nicolet 6700 FTIR spectrometer equipped with an attenuated total reflectance (ATR) accessory. The measurements were taken over a wavenumber range of 400 to 4000 cm^–1^, with 64 scans at a resolution of 4 cm^–1^.

Rheological characterization of the samples was used as the second response for the DoE. These tests were conducted on an Anton Paar MCR 302 rheometer. Dynamic strain sweep tests were carried out using a 25 mm parallel plate (PP-25 mm) geometry under a nitrogen atmosphere at 200 °C to determine the linear viscoelastic region (LVE). The tests were performed at a frequency of 1 rad/s with a strain range of 0.01–100%, and the LVE region was set at 3% strain. Additionally, small-amplitude oscillatory shear tests were performed in the LVE over a frequency range of 0.01 to 500 rad/s, using the same PP-25 mm geometry and a 1 mm gap, also at 200 °C.

Scanning electron microscopy and energy-dispersive X-ray spectroscopy (SEM-EDS) images were obtained by using a FEG-TESCAN MIRA microscope operating at 20 keV and 1 nA. All samples were coated with gold (Au).

Electron paramagnetic resonance (EPR) spectroscopy analysis was done using an EPR spectrometer (Bruker, Germany) operating at a power of 20 dB, a center field of 3514 G, and a sweep width of 100 G at room temperature.

### 2.5 Sample Preparation for EPR

Suspensions of FLG, Irganox B215, and TiO_2_ in deionized (DI) water with concentrations of 0.2, 0.1, and 0.3 mg/mL, respectively, were prepared using an ultrasonic bath operating at a frequency of 60 kHz for 60 min. The suspensions were then added to a mixture of H_2_O_2_ (50 mmol/L) and DMPO (50 mmol/L), which served as a spin trapper, and exposed to UV–C light using one commercial Hg lamp (Philips TUV 4 W) emitting light at a peak wavelength of 254 nm (471 kJ/mol) for 5 min. The same procedure was followed for the mixture of 0.3 mg/mL TiO_2_ with 0.2 mg/mL FLG and the mixture of 0.3 mg/mL TiO_2_ with 0.1 mg/mL Irganox B215. It is important to note that, under UV–C exposure, H_2_O_2_ undergoes photolysis and chemical bond cleavage, generating OH radicals that form DMPO–OH adducts, which are detectable by EPR.^18^

### 2.6 Design of Experiments (DoE)

To achieve optimal photoprotection and evaluate whether FLG or Irganox B215 (B215) synergistically enhances photoprotection when combined with TiO_2_, we employed a Design of Experiments (DoE) approach. Specifically, a 2^2^ modified factorial design with a central point was used to process the composites.^31^

In this study, the variables tested were the mass percentages (%m/m) of TiO_2_, FLG, and Irganox B215, which were combined into pairs as TiO_2_ + FLG and TiO_2_ + Irganox B215. Tables 1 and 2 outline the levels and corresponding %m/m for each stabilizer and detail the 2^2^ modified factorial design, the nomenclature used, and the experimental results obtained for each response variable in this study.

**Table 1:**
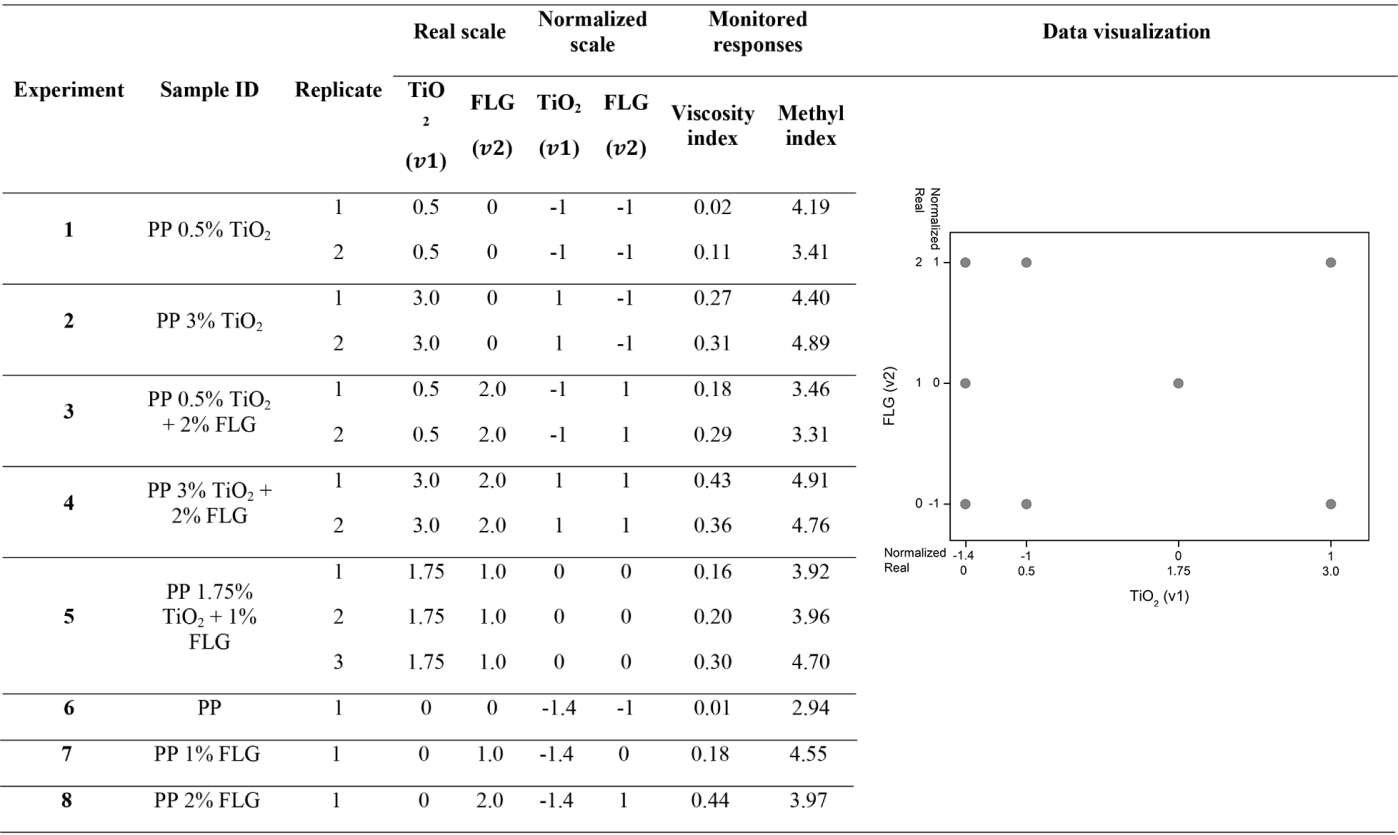
Modified Factorial Planning Design for the Samples with Titanium Dioxide and Few-Layered Graphene

**Table 2:**
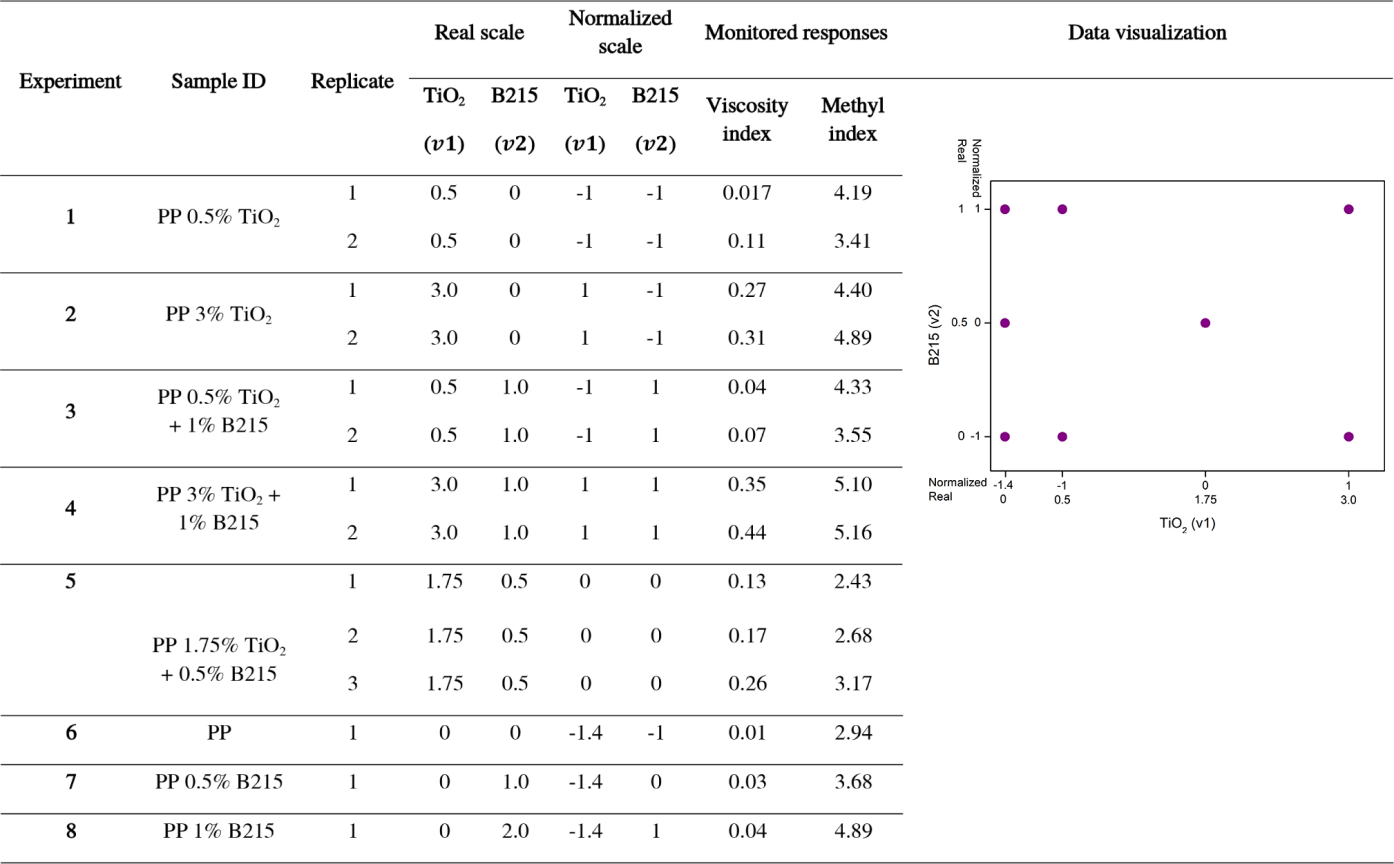
Modified Factorial Planning Design for the Samples with Titanium Dioxide and Irganox B215

The responses used for the TiO_2_ + FLG and TiO_2_ + Irganox B215 designs were the methyl index (MI)^32^ and the zero-frequency complex viscosity *r*, derived from rheological data. The methyl index was calculated as the ratio of the deconvoluted area of the FTIR-ATR peak at 1456 cm^–1^ to the reference peak at 1170 cm^–1^. This deconvolution was performed using Voigt profile fitting, which results from the convolution of Gaussian and Lorentzian functions.^33^ The Voigt profile is commonly applied in the analysis of spectroscopy data from techniques such as X-ray diffractometry and infrared spectroscopy.^31,34^

The methyl index (MI) is associated with the release of volatile compounds containing CH_3_ groups during photodegradation. The release of these compounds reduces the intensity of the FTIR-ATR peak at 1456 cm^–1^, indicating that a higher MI corresponds to a lower release of volatiles resulting from photodegradation.^32^ The viscosity index (eq 1) reflects the extent of chain scission occurring during UV-C photodegradation of polypropylene. Consequently, higher viscosity index values correspond to lower levels of chain scission during photodegradation.

1 $$\mathrm{Viscosity}\;\mathrm{Index}=\frac{\eta_{0\mathrm{sample}96h}}{\eta_{0\mathrm{sample}0h}}$$

The experiments outlined in Tables 1 and 2 enabled us to employ a response surface methodology (RSM) to evaluate the behavior of each variable, *v*1 (%m/m of TiO_2_) and *v*2 (%m/m of Irganox B215 or %m/m of FLG), and one second-order interaction effect, *v*1*v*2, on the viscosity index and methyl index independently, and within a global statistical model. This global model was built using the desirability function (*D*), as proposed by Derringer and Suich.^35^ In this approach, individual responses are transformed into a dimensionless scale (*d_i_*), calculated according to eq 2,^36^ which ranges from 0 (for an unacceptable response value) to 1 (for an acceptable response value). The overall desirability (*D*) is calculated as the geometric mean of the individual desirability values (*d_i_*) for each response.^31^ In this study, the optimal condition for achieving high photoprotection was identified when both the methyl index and the viscosity index desirability reached their maximum values, i.e., 1.

2 $$d_{i}=f\left(x\right)=\{\begin{array}{l} 0,\mathrm{if}\;y<L\\ {\left(\frac{y-L}{T-L}\right)}^{s},\mathrm{if}\;L\leq y\leq T\\ 1,\mathrm{if}\;y>T \end{array}$$

In eq 2, *y* is the measured response, *L* is the accepted response, i.e., the lowest response obtained from the viscosity index and methyl index, *T* is the target response, i.e., the highest response from the viscosity index and methyl index, and *s* is the weighting factor for each response (in this case, *s* = 1). The optimized global response was calculated by using the geometric mean of the methyl index and viscosity index. The resulting RSM matrix was analyzed by using Octave (open-source software) and Excel.

## 3 Results and Discussion

### 3.1 Morphological Characterization of the Composite Samples by SEM-EDS

Figure 1 shows the SEM and SEM-EDS images for the mixture of 3% (by mass) TiO_2_ with 2% FLG, as well as the sample containing 3% TiO_2_. It is well-known that the dispersion and distribution of stabilizers/additives within the polymer matrix play a crucial role in determining the composite’s properties, particularly in relation to photoprotection.^18^ As shown in Figure 1a and b, both TiO_2_ and FLG are distributed well within the PP matrix. However, the interfacial adhesion between the stabilizers and the polymer appears to be weak. The SEM-EDS image (Figure 1e) also indicates that the TiO_2_ stabilizer has poor dispersion within the polymer matrix. The same trend was observed for the sample containing 3% TiO_2_, as shown in Figure 1c, d, and f. To improve adhesion and compatibilization, surface functionalization of TiO_2_ by silane can be used.^37^ To minimize the influence of any functionalization of TiO_2_’s surface on the effect of FLG or B215 in radical scavenging, not only PP radicals but also TiO_2_’s electron–hole parallel reactions, this study focused on exploring these mixtures without any surface modification.

**Figure 1:**
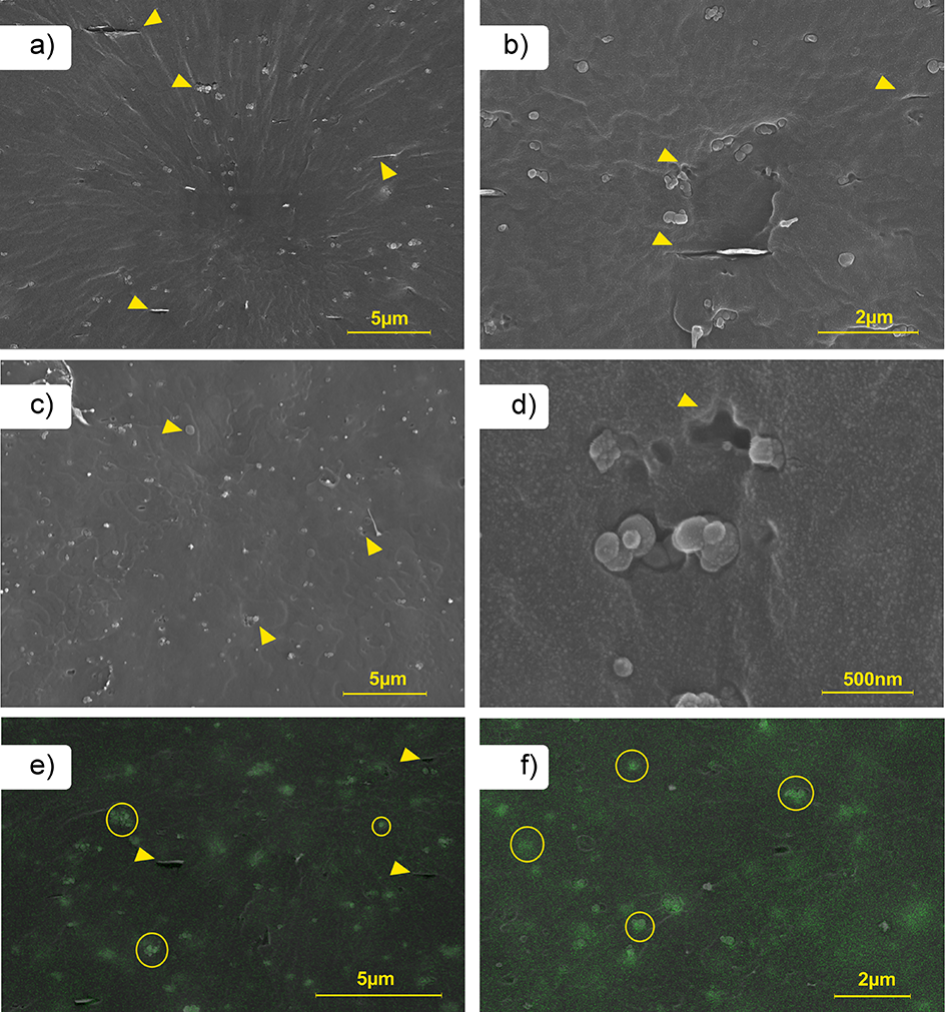
SEM images of PP composite samples without UV-C exposure for the mixture of 3% TiO_2_ with 2% FLG, with a magnification of (a) 10k× and (b) 30k×; for 3% TiO_2_, with a magnification of (c) 10k× and (d) 110k×. SEM-EDS images for (e) 3% TiO_2_ + 2% FLG and (f) 3% TiO_2_ samples.

#### 3.1.1 Effect of the Mixture of FLG with TiO_2_ and TiO_2_ with B215 in the Polypropylene Matrix in FTIR and Rheology

The responses used to build the Design of Experiments (DoE) models are presented in Figures 2 and 4, where Figure 2a shows a typical FTIR transmittance spectrum for neat PP without any UV–C exposure. At the same time, Figure 2b displays the spectra for composites containing TiO_2_ and FLG, measured at 0 h and after 96 h of UV-C exposure. The inset highlights the effect of UV-C exposure on the peak at 1456 cm^–1^, which corresponds to the asymmetric bending of C–H bonds in methyl groups.^25,27,32^ A decrease in the intensity of this peak is observed across all samples after UV-C exposure, regardless of the additive used. Figure 2c illustrates the methyl index (MI) for all samples before and after UV-C exposure.

**Figure 2:**
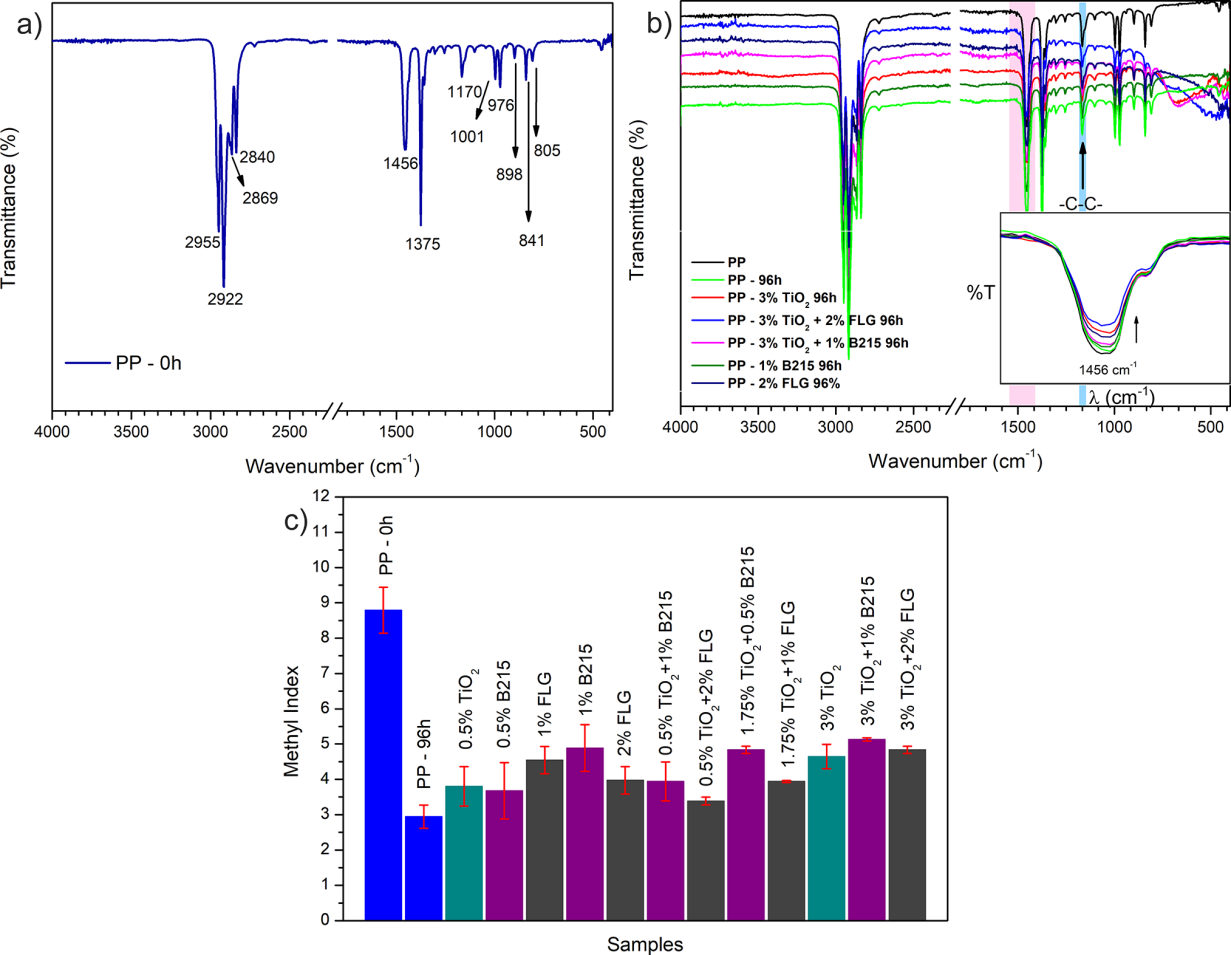
(a) Typical FTIR spectra for neat PP without UV-C exposure, (b) FTIR spectra for PP samples with 3% TiO_2_, 3% TiO_2_ + 2% FLG, 3% TiO_2_ + 1% B215, 2% FLG, and 1% FLG before and after 96 h of UV-C irradiation, and (c) methyl index for neat PP prior and after 96 h of UV-C exposure and for all samples after 96 h of UV-C exposure.

**Figure 3:**
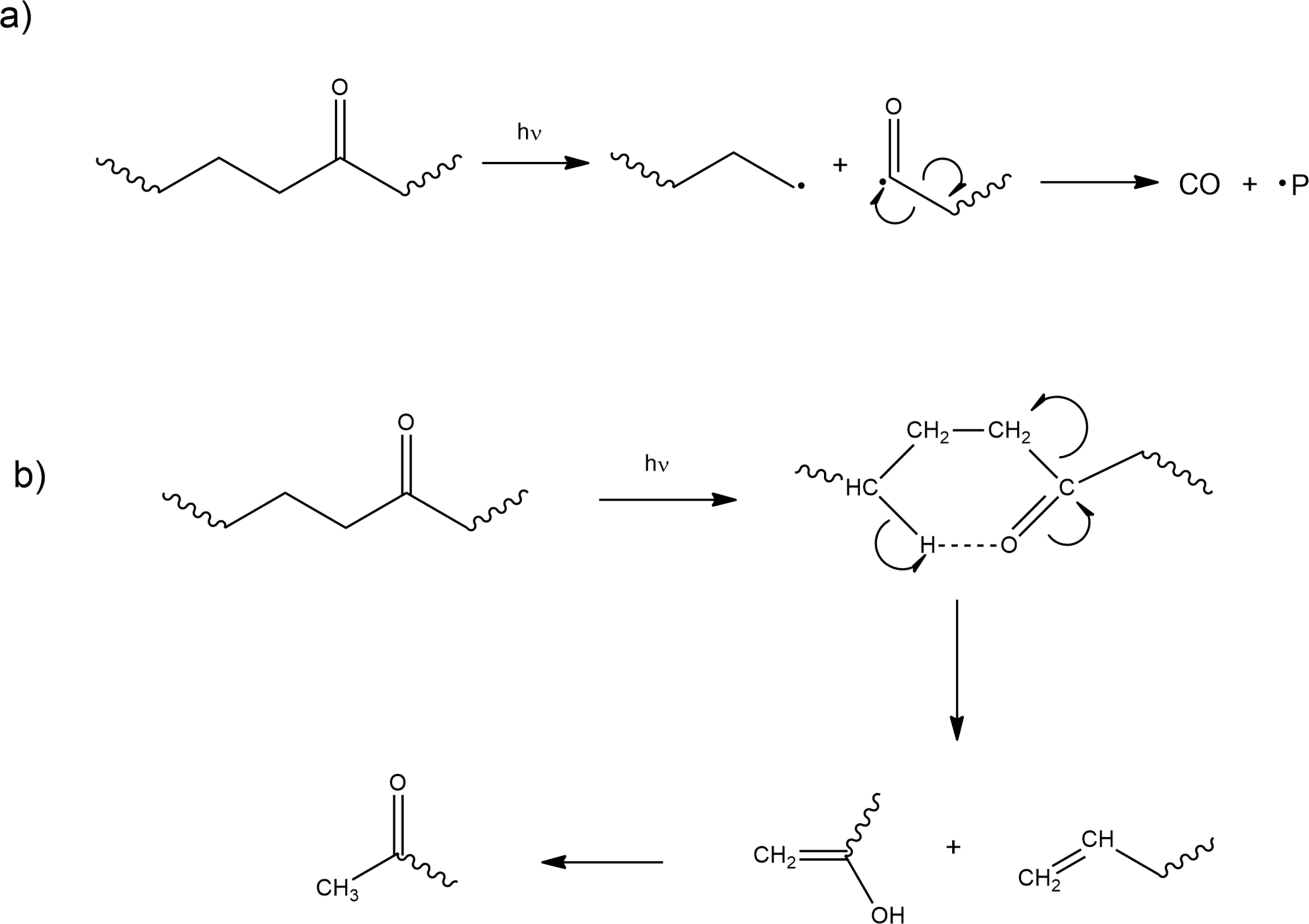
(a) Norrish Type I and (b) Norrish Type II mechanisms.

Table 1 summarizes the viscosity and MI of TiO_2_ + FLG samples, and Table 2 summarizes those of TiO_2_ + B215 samples. The sample with 3% TiO_2_ + 1% B215 presented the highest MI among all samples analyzed, suggesting that adding B215 was more effective in protecting PP against the release of volatiles containing −CH_3_ moieties.^32^ Being a small molecule, B215 has a high dispersion within the polymer matrix during extrusion. In the case of sample PP 3% TiO_2_ + 2% FLG, the effect observed may be attributed to the 2D structure of FLG and its high surface area, which acts as a physical barrier within the polymer matrix, creating tortuous pathways that hinder the diffusion of small molecules,^6,38^ limiting the release of volatiles. Overall, UV-C exposure resulted in a decrease in MI values for all samples after 96 h. As shown in Figure 2c, the addition of FLG to TiO_2_ had a small influence on the evolution of MI during UV-C photodegradation; the same behavior was observed in the samples containing Irganox B215, i.e., the variable that influences MI the most is the %m/m of TiO_2_ in both cases.

The carbonyl group is a result of PP photodegradation. The presence of these groups enhances PP’s UV sensitivity, leading to further photodegradation reactions through Norrish Type I and II reactions (Figure 3).^27^

In a previous study,^39^ the carbonyl peak only became detectable after 96 h of UV-C exposure. Therefore, the MI proposed by Rouillon et al.^32^ is a reliable method not only for assessing the early stages of photodegradation but also for this specific exposure time frame. This is evidenced by the decrease in the methyl peak, resulting from the release of volatile compounds containing −CH_3_ groups: after 25 h of irradiation for UV-A and UV-B, as observed by Rouillon et al.,^32^ and in less than 10 h for UV-C.^39^

Figure 4 displays the complex viscosity as a function of the angular frequency for experiments 1, 4, 5 (CP), and 6, as outlined in Tables 1 and 2, after 96 h of photodegradation, as well as for neat PP before and after UV-C exposure. Figure 4a shows the results for the TiO_2_ + FLG combination, while Figure 4b presents the data for the TiO_2_ + B215 combination. The results of complex viscosity for all samples, before UV-C exposure, are available in the Supporting Information and Figure S3.

**Figure 4:**
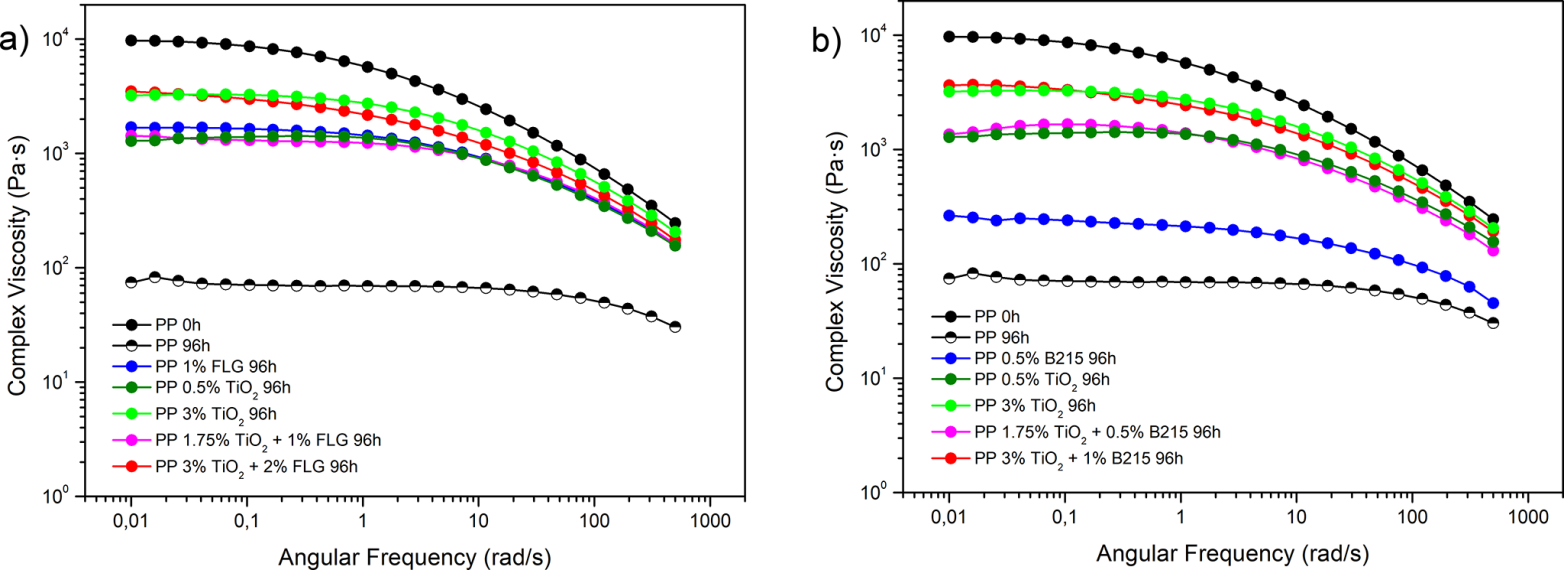
Complex viscosity as a function of the angular frequency for experiments 1, 4, 5 (CP), and 6 after 96 h of photodegradation, where (a) shows the data for PP composites of (a) TiO_2_ with FLG and (b) TiO_2_ with B215.

It is shown in Figure 4 that the reduction in complex viscosity of the composites after 96 h of UV–C photodegradation is less pronounced in the composites than in neat PP. Compared to samples containing only FLG or B215, mixing TiO_2_ with B215 or FLG resulted in a smaller decrease in complex viscosity. However, in the samples where TiO_2_ was mixed with FLG, the reduction in complex viscosity, after 96 h of UV–C photodegradation, was less pronounced than in the samples mixed with B215, suggesting low chain scissions in samples containing FLG. Table S1 summarizes the percentages of reduction from the complex viscosity curves for each composite and combination.

The observed results can be explained by the different photoprotection mechanisms of the various additives. Figure 5 illustrates the autocatalytic photodegradation cycle of the PP, highlighting the points at which each photostabilizer acts. While Irganox B215 functions as both a primary and secondary antioxidant, scavenging radicals generated during the PP photodegradation process, FLG can act as a UV screener/absorber as well as a radical scavenger,^18^ contributing more to the photoprotection. TiO_2_ primarily functions as a UV screener/absorber, blocking light before the polymer absorbs it and becomes an excited molecule. This outcome is also observed in the RSM obtained from the DoE, as shown in the section *Few-Layered Graphene and Irganox B215 DoE*.

**Figure 5:**
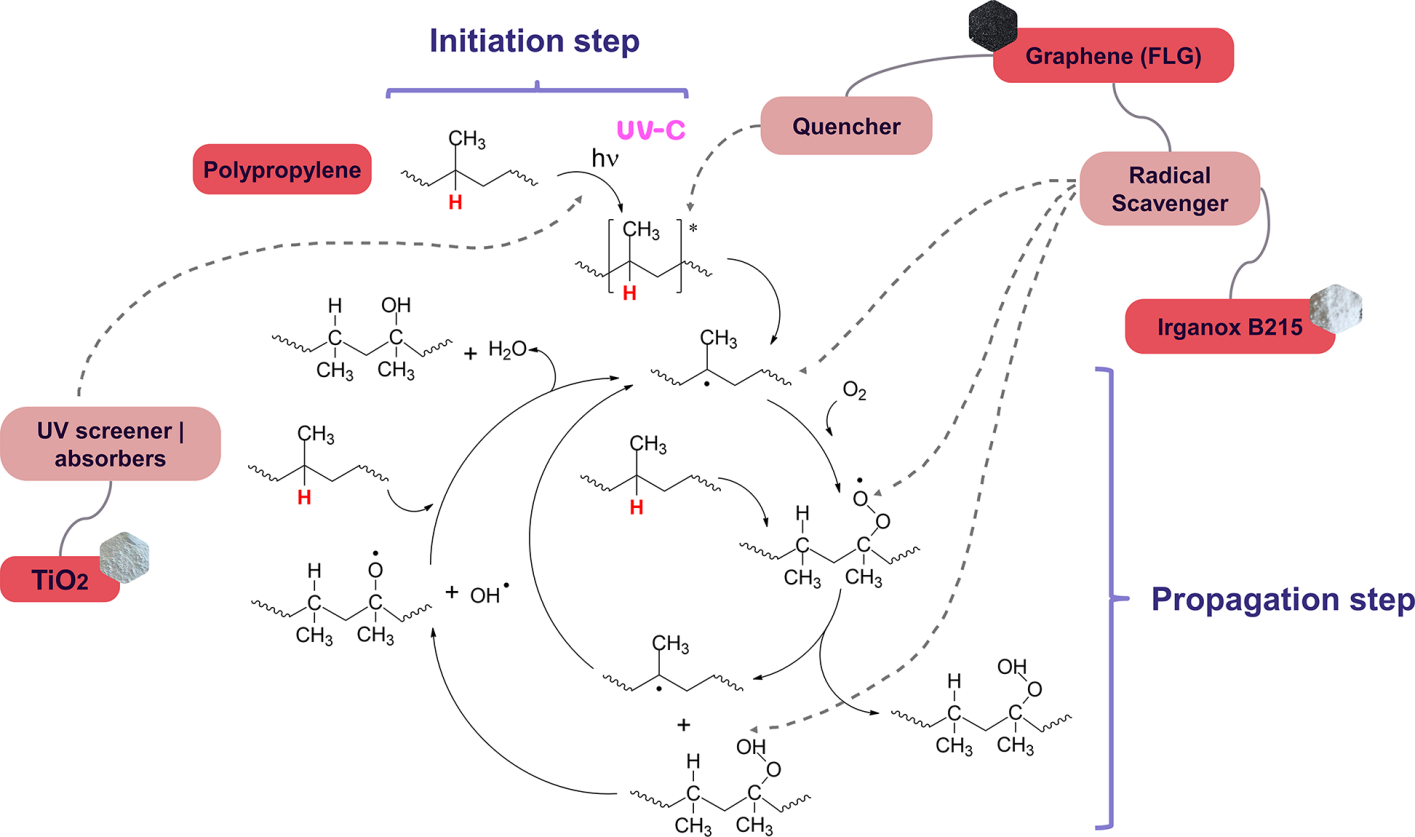
Autocatalytic photodegradation cycle and photostabilizers’ mechanism.

The UV-induced photodegradation of PP has been extensively studied in the literature.^25,27,32,40^ This process involves two competing reactions: cross-linking (or branching) and chain scission. Both reactions directly affect the polymer’s molar mass and distribution, consequently influencing its properties. Chain scission is an expected outcome of PP photodegradation, even under UV-C exposure, leading to a loss in pseudoplastic behavior after 96 h of UV-C photodegradation, as shown in Figure 4, and is associated with a reduction in molar mass. This reduction, in turn, leads to significant losses in the polymer’s mechanical and physicochemical properties.

### 3.2 Few-Layered Graphene and Irganox B215 DoE

TiO_2_ is widely used as a photostabilizer due to its action as a UV absorber or screener to shield polymers from UV radiation;^8–11^ however, it can also generate reactive oxygen species (ROS), which can attack polymer molecules, promoting further degradation processes.^24–27^ Thus, combining TiO_2_ with a radical scavenger can be a promising alternative to enhancing polymer photostabilization.

In this context, Figure 6 shows the response surface model (RSM) generated by the design of experiments (DoE) for each mixture of TiO_2_ with FLG or B215, illustrating the methyl index (MI) and viscosity index responses illustrates the response surface model (RSM) generated by the design of experiments (DoE) for each mixture of TiO_2_ with FLG or B215, showing the responses to the methyl index (MI) and viscosity retention separately. Tables S1–S6 present the results of ANOVA for each RSM, and Table S7 summarizing the *p*-values, *F* Critical, and *F* calculated values for all DoE with α = 0.01 and α = 0.05 can be found in the Supporting Information.

**Figure 6:**
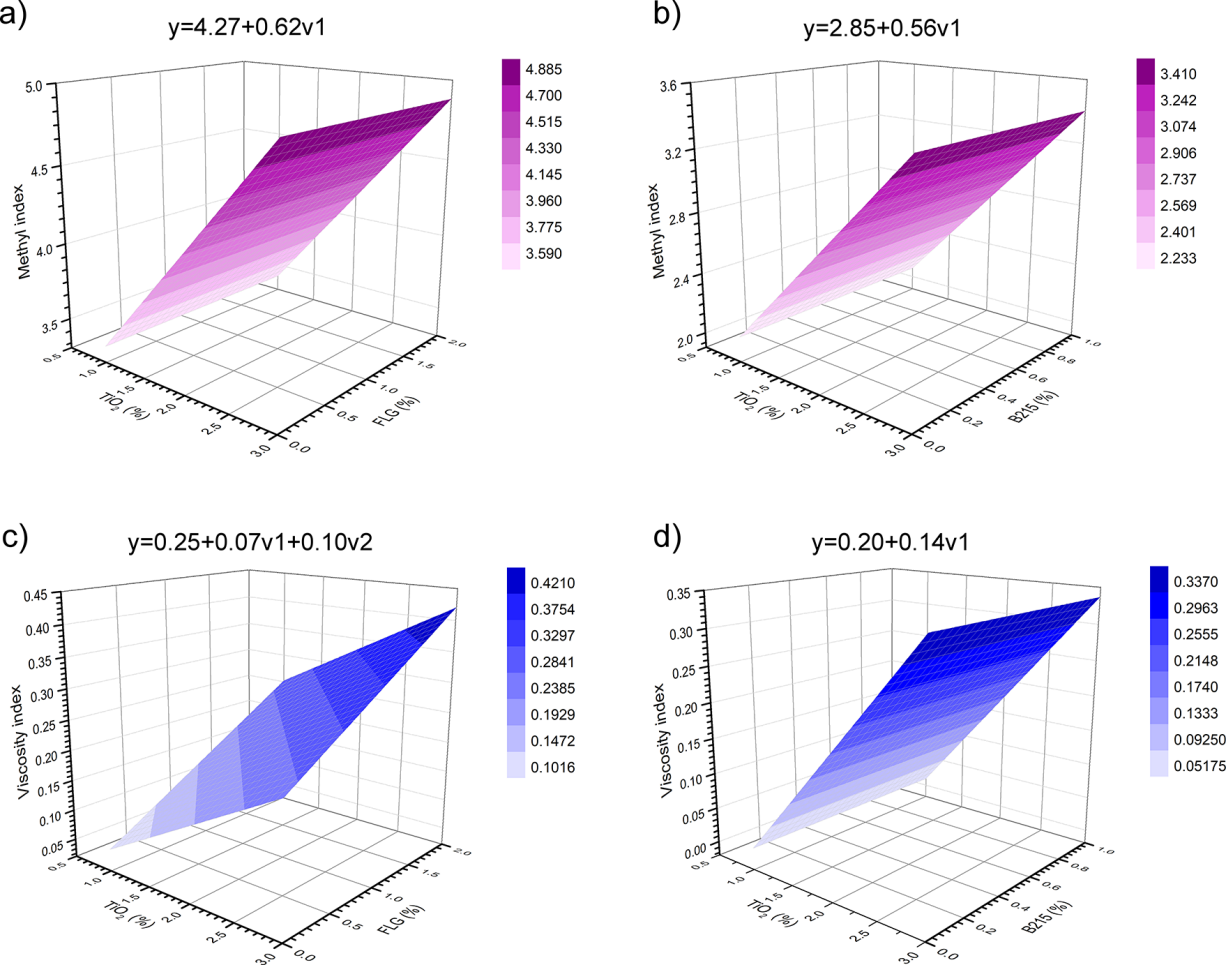
RSM for the methyl index (MI) responses for (a) TiO_2_ + FLG and (b) TiO_2_ + B215 samples, as well as the viscosity index for the (c) TiO_2_ + FLG and (d) TiO_2_ + B215 samples.

The MI was selected as a response variable to monitor the photodegradation of PP. This index was first proposed by Rouillon et al.^32^ and serves as an alternative method to track PP photodegradation under UV-A and UV-B using FTIR, replacing the traditional carbonyl index. The authors detected volatile species containing −CH_3_ groups within 25 h of photodegradation and noted that the release of these species decreased the area of the peak at 1456 cm^–1^ in the FTIR spectra. Additionally, this index has been shown to be effective in tracking photodegradation even under UV-C exposure.^39^

The response surface for the mixture of TiO_2_ with FLG (Figure 6a) or with B215 (Figure 6b) on MI indicates that only the %m/m of TiO_2_ (*v*1) is responsible for impacting this response, i.e., the %m/m of TiO_2_ (*v*1) is responsible for decreasing the release of volatile compounds during the PP UV-C photodegradation. It is worth noting that in Figure 2b, the MI values are higher than those in Figure 6a, suggesting that adding FLG was more effective than adding B215 for this response.

It is known that graphene can act through various polymer photostabilization mechanisms, such as UV absorbers/screeners, physical barriers, and radical scavengers.^6^ In this case, the effect observed in Figure 6b may be attributed to the 2D structure of FLG and its high surface area, which acts as a physical barrier within the polymer matrix, inhibiting the diffusion of low-molecular-weight compounds by creating tortuous pathways that hinder the diffusion of small molecules, such as oxygen, and free radicals into the polymer bulk.^6,38^ Therefore, in this case, FLG may also be working as a physical barrier, reducing the input of O_2_ within the PP matrix, slowing photooxidation, and limiting the release of volatiles.

As is already known in the literature, chain scission is a typical consequence of PP photodegradation and is directly associated with the complex viscosity.^25,27,32,40^ To indirectly evaluate the impact of UV-C exposure on PP chain scission, the viscosity index was used, comparing the photodegraded sample to the nonexposed sample. Lower viscosity index values indicate that the polymer underwent fewer chain scission reactions.

In the case of the RSM for the viscosity index response, the mixture of TiO_2_ with FLG (Figure 6c) exhibited a different behavior, as observed for the MI response. Both individual variables, the %m/m of TiO_2_ (*v*1) and the %m/m of FLG (*v*2), as well as their interaction (*v*1*v*2), significantly influenced the viscosity index. The best results, i.e., viscosity index with high values, were achieved when both *v*1 and *v*2 were at their highest levels (+1) in the DoE.

This result suggests that the addition of FLG to TiO_2_ reduced chain scission during the photodegradation of PP under UV-C. This effect may be attributed not only to the combination of TiO_2_’s photoprotection mechanism as a UV screener/absorber with FLG, but also to FLG’s distinct photoprotection mechanism, which includes acting as a UV screener/absorber and radical scavenger, as well as serving as a gas barrier due to its high 2D surface area.^6,17,18,41^ FLG may not only scavenge radicals generated during PP photodegradation but also capture radicals produced by TiO_2_, showing a synergistic effect. The mixture of TiO_2_ with B215 (Figure 6d) revealed that the %m/m of TiO_2_ (*v*1) was the primary variable influencing the viscosity index response. In this case, the addition of Irganox B215 (*v*2) to TiO_2_ did not significantly affect this response.

Additionally, a global statistical model, shown in Figure 7, was used to evaluate the impact of the variables, the %m/m of TiO_2_ (*v*1), and the %m/m of FLG (Figure 7a) or %m/m of B215 (Figure 7b) (*v*2), as well as their interaction (*v*1*v*2), when combining both responses, methyl index, and viscosity index in the PP photoprotection against UV-C. The responses were combined using the desirability function (*D*) proposed by Derringer and Suich.^35^

**Figure 7:**
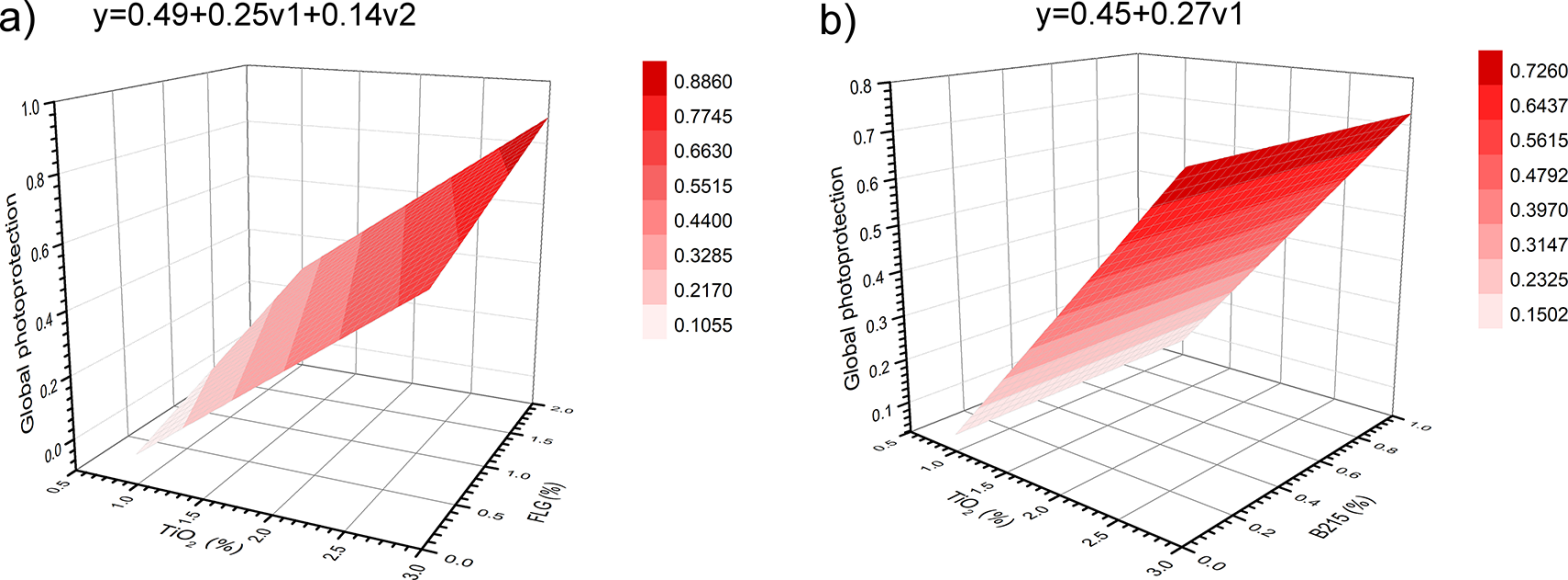
Global statistical model for (a) TiO_2_ + FLG and (b) TiO_2_ + B215 samples.

Figure 7a shows the global statistical protection model for the mixture of TiO_2_ and FLG, and Figure 7b shows the global statistical protection model for the mixture of TiO_2_ and Irganox B215. It is possible to observe that adding FLG to TiO_2_ not only led to higher values of photoprotection than adding B215 to TiO_2_ but also showed that the global model is impacted by the %m/m of FLG, suggesting a possible synergistic effect between them. As said previously, this effect may be due to the combination of the different FLG photoprotection mechanisms and its high 2D surface area, which provides more active sites for scavenging radicals from PP photodegradation and TiO_2_’s parallel reactions under UV light than Irganox B215. Thus, to achieve better photoprotection against UV-C, it is suggested to work with both %m/m TiO_2_ and %m/m FLG at the level +1 in this DoE, i.e., 3% m/m TiO_2_ and 2% m/m FLG.

### 3.3 Electron Paramagnetic Resonance (EPR)

Electron paramagnetic resonance (EPR) was used to evaluate the effect of FLG and Irganox B215 as radical scavengers in a mixture with TiO_2_. The EPR spectra obtained from spin-trapping with DMPO after 5 min of UV-C irradiation in deionized water suspensions of FLG, TiO_2_, Irganox B215, and the mixtures of TiO_2_ with FLG and with Irganox B215 are shown in Figure 8. The spectra display a characteristic 1:2:2:1 quartet signal, indicating the presence of the DMPO–OH adduct after UV-C exposure.^42^ Upon irradiation, the signal intensity corresponding to the TiO_2_ suspension increased, suggesting that reactive oxygen species (ROS), i.e., −OH groups, may be generated on the TiO_2_ surface after exposure to UV-C.^43^ This signal intensity increased by around 21% compared to the control (DMPO + H_2_O_2_).

**Figure 8:**
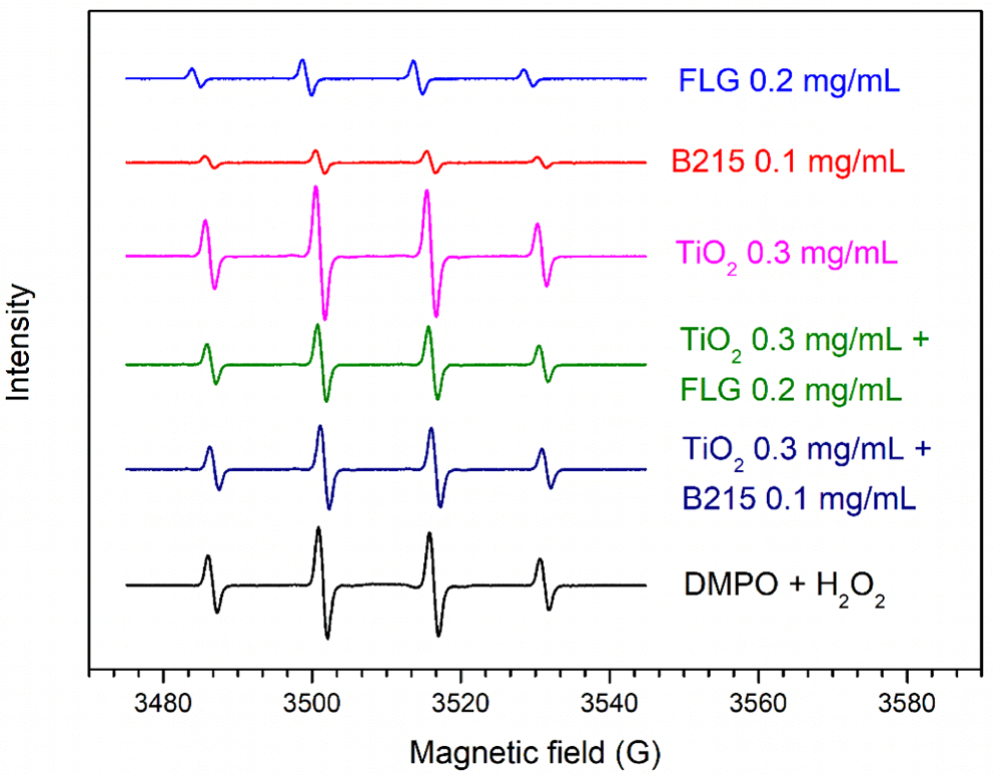
EPR spectrum obtained from spin-trapping with DMPO after 5 min of UV-C irradiation in a deionized water suspension of FLG, TiO_2_, Irganox B215, and the mixing of TiO_2_ with FLG and with Irganox B215.

The absorption region of TiO_2_ ranges from 200 to 400 nm, with a peak at around 345 nm and an absorption edge of about 360 nm.^23,44^ Although rutile TiO_2_ exhibits low photocatalytic activity due to its bandgap value of 3.03 eV,^23^ it still generates electron–hole pairs in the presence of water. These electron–hole pairs on the TiO_2_ surface lead to reduction and oxidation reactions (Figure 9), respectively, generating reactive oxygen species (ROS)^22^ as parallel reactions, which can further promote photodegradative processes in polypropylene.

**Figure 9:**
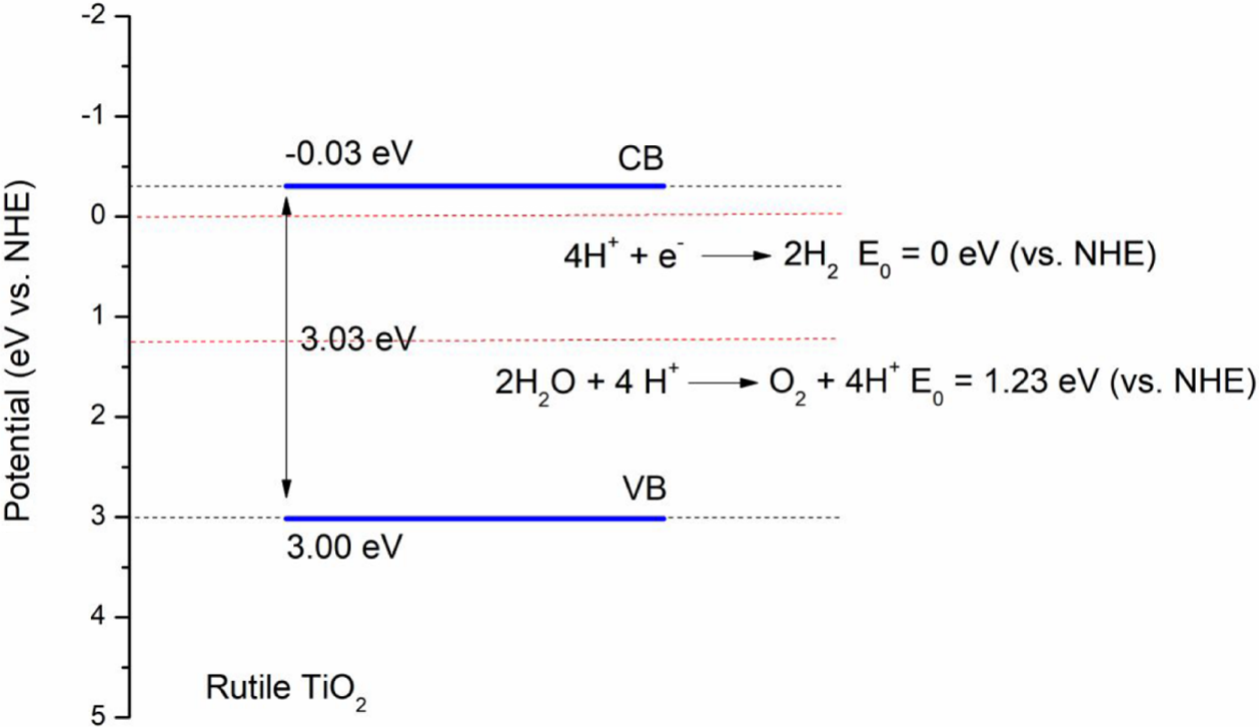
ROS formation reactions on the TiO_2_ surface.

The addition of radical scavengers can help mitigate the formation of reactive oxygen species by TiO_2_. The EPR signal intensity (Figure 8) of Irganox B215 decreased significantly by approximately 79% compared with the control. This behavior can be attributed to the composition of the Irganox B215 blend, which consists of a 2:1 ratio of Irgafos 168, an organophosphate that decomposes peroxide radicals, and Irganox 1010, an H-donor.^12,45^ These compounds function by scavenging −OH• radicals, thereby decreasing the number of free hydroxyl radicals that can form adducts with DMPO.^5,18,46^

The phosphorus atom in Irgafos 168 reacts with oxygen species, increasing its oxidation state from +3 to +5, thereby scavenging radicals. In addition to being an H-donor, sterically hindered phenols, such as Irganox 1010, are known to inhibit the autoxidation of polymers by undergoing numerous further chemical reactions. After hydrogen abstraction, the phenoxyl radical reacts with a hydroxyl radical,^46^ which can be seen in the decrease in the EPR signal intensity (Figure 8). In PP, hydroperoxides are formed through hydrogen abstraction by a peroxy radical from the polyolefin polymer backbone (Figure 5).^47^

Another radical scavenger that has gained attention as an alternative photostabilizer in polymers is graphene and its derivatives.^13–19^ As shown in Figure 8, FLG also reduces the EPR signal intensity by around 68%, effectively scavenging the radicals. Karimi et al.^18^ demonstrated that 57% of the total reduction in the EPR signal intensity can be attributed to FLG’s UV absorber/screener effect, while 43% can be due to its free radical scavenging capability.

Nevertheless, when comparing the mixture of TiO_2_ with Irganox B215 and the mixture of TiO_2_ with FLG, the reduction in the EPR signal intensity (Figure 8) was greater for the TiO_2_ + FLG mixture (∼30%) than for the TiO_2_ + B215 mixture (∼25%). This difference may be attributed not only to FLG’s distinct photoprotection mechanisms but also to its high 2D surface area, which provides more active sites for scavenging radicals generated from TiO_2_’s parallel reactions compared to Irganox B215. These results are in agreement with the RSM for the mixture of TiO_2_ with FLG or B215, as shown in Figure 7.

Table 3 summarizes the influence of each stabilizer and how it impacts each response (viscosity index and methyl index) used in this work. Even though the stabilizers had poor adhesion and dispersion, they were agglomerated, as indicated by SEM-EDS, and the RSM showed that the mixtures of TiO_2_ + FLG and TiO_2_ + B215 are effective in photoprotecting PP against UV-C. RSM data showed that TiO_2_ is the primary variable directly affecting the evolution of the methyl index. EPR showed that when TiO_2_ absorbs UV-C radiation in the presence of water and oxygen, it increases reactive oxygen species (ROS) levels. In the polymer matrix, this leads to parallel reactions that may promote further PP degradation. In contrast, FLG proved effective in reducing chain scission, as shown by the rheological analysis, and, when combined with TiO_2_, showed a synergistic effect, improving PP photostabilization. Irganox B215 demonstrated efficacy as a radical scavenger, as shown by EPR; however, when mixed with TiO_2_, the EPR and RSM showed that it was unable to scavenge radicals generated by TiO_2_’s parallel reactions. Additionally, the RSM and MI showed that Irganox B215 had a minimal impact on the evolution of the methyl index.

**Table 3:** Summarized Results for Each Stabilizer – TiO_2_, FLG, and Irganox B215 – and Each Response – Viscosity Index and Methyl Index – from DoE

	TiO_2_	FLG	Irganox B215
Chain scission—viscosity index	Increases ROS levels, promoting chain scission through parallel reactions.	High 2D surface area provides more active sites for radical scavenging, reducing chain scission.	Effective antioxidant action against •OH radicals, minimizing chain scission.
FTIR-ATR—methyl index (MI)	Strong UV-screening effect significantly impacts the evolution of MI.	Hinders diffusion of small molecules, slowing photo-oxidation and limiting volatile release, affecting MI.	Minimal influence on MI evolution.
Photoprotection mechanism	UV screener/absorber	UV screener/ absorber, radical scavenger, physical barrier	Radical scavenger

## 4 Conclusion

In this study, the influence of few-layered graphene and Irganox B215 when combined with titanium dioxide (TiO_2_) for UV-C photoprotection of polypropylene was investigated. The use of two different types of photostabilizers was employed not only to reduce radical formation caused by photodegradation but also to mitigate radicals generated by the electron–hole reactions of TiO_2_. To achieve this, we employed a Design of Experiments (DoE) as a tool to (i) maximize the responses and (ii) minimize the number of experiments, thereby optimizing the outcomes.

The surface response analysis indicated that unlike B215, the addition of FLG had a synergistic effect with TiO_2_, further improving photoprotection. This may be attributed to the combined photoprotection mechanisms of FLG, which act not only as a radical scavenger but also as a UV screener/absorber, thereby reducing radical formation from both the PP photodegradation cycle and the electron–hole generation from TiO_2_.

EPR results showed that mixing radical scavengers with TiO_2_ reduced the OH formation by ∼30% for the FLG and ∼25% for the B215 mixture, and although SEM images showed that the stabilizers had poor dispersion but good distribution, the DoE showed that the addition of FLG had a synergistic effect with TiO_2_, and working on the highest level (+1), i.e., % m/m, it enhanced the PP UV-C photoprotection, diminishing chain scission and scavenging TiO_2_ ROS, thereby enhancing UV-C resistance in PP.

## Supplementary Material



## Data Availability

Data will be made available upon request.

## Notes

Detailed XRD, Raman, and SEM analyses of FLG and TiO_2_, as well as complex viscosity data for all samples before UV-C exposure; a table summarizing the percentage reduction in complex viscosity for each sample, and ANOVA tables for each RSM model (PDF)
