# Supplementary material for: Mitigating UV‑C Degradation in Polypropylene Using Hybrid TiO2/Few-Layer Graphene/Photostabilizer Systems
Source: ACS Omega. 2025 Oct 27;10(44):53467–78. doi: 10.1021/acsomega.5c08936 (PMC12612903; doi:10.1021/acsomega.5c08936)
Supplement: Supplementary file 1 [file ao5c08936_si_001.pdf]

Supporting Information

Mitigating UV-C Degradation in Polypropylene Using Hybrid TiO_2/Few-Layer Graphene/Photostabilizer Systems

Jessica C. F. Gimenez[1,3], Robert Paiva[1], Sophia H. F. Bonatti[1], Lucas H. Staffa[2], Edenir R. Pereira-Filho [1], Emna Helal[3,4], Nicole R. Demarquette[3], Manoel G. P. Homem[1], Sandra A. Cruz[1*].

[1] Department of Chemistry, Exact Sciences and Technology Centre (CCET), Federal University of São Carlos (UFSCar), Rodovia Washington Luís, Km 235, 10 SP-310, São Carlos, Brazil

[2] Department of Materials Engineering, Exact Sciences and Technology Centre (CCET), Federal University of São Carlos (UFSCar)

[3]Mechanical Engineering, École de Technologie Supérieure (ÉTS), 1100 Notre-Dame St W, H3C 1K3 Montréal, Quebec, Canada

[4]NanoXplore Inc., Saint-Laurent, Quebec, Canada

KEYWORDS: Polypropylene, graphene, composite, UV-C, photostabilization, few-layered graphene, titanium dioxide

SUPPORTING INFORMATION

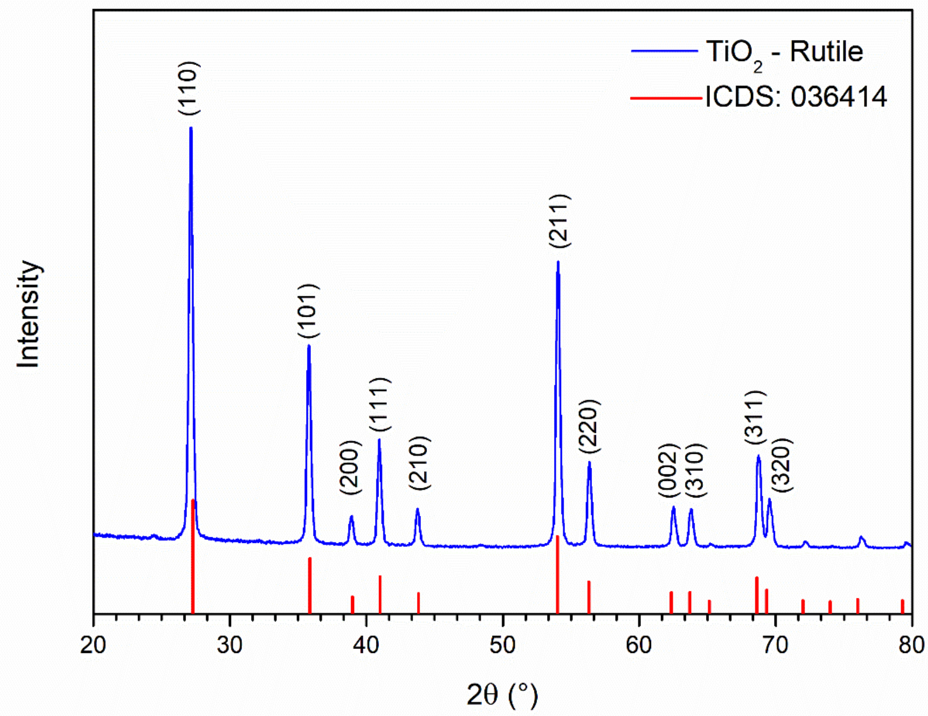

Figure S1. XRD patterns of nano-TiO_2 in the rutile phase (ICDS 036414).

TiO_2 crystalline phase was identified by X-ray diffraction (XRD) using an XRD-6100 SHIMADZU, at 40 kV with Cu Kα radiation (λ=1.54 Å), step length of 0.02°, scan rate of 10° min^{-1}, and scan range from 10 to 80° θ.

Figure S4 shows the XRD for nano-TiO_2 in the rutile phase. The X-ray diffractogram shows a pattern that matches ICDS: 036414. It is possible to verify the characteristic peaks for TiO_2 in the rutile phase (110), (101), (200), (111), (210), (211), (220), (002), (310), (311), and (320).

The rutile phase presents a bandgap of 3.03 eV, and it is known for being less efficient than the anatase phase as a photocatalyst. It exhibits strong absorption between 260-330 nm, with a peak at 308 nm, and a refractive index of 2.73, which is higher than that of

polypropylene (1.503). With a particle size lower than 100 nm, it can be used as a screener against UV radiation in polymers.

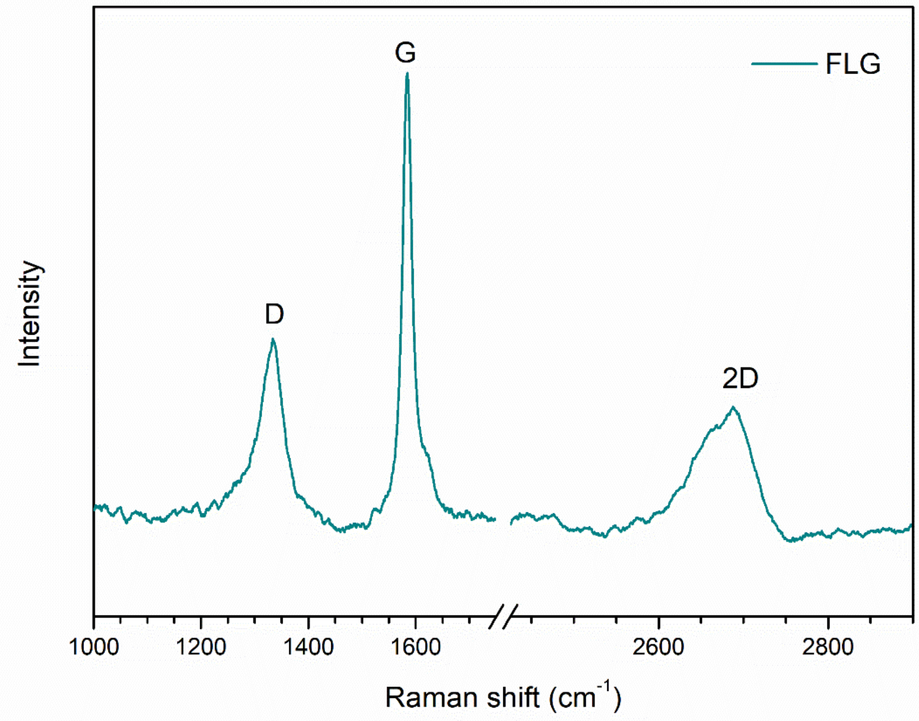

Figure S2. Raman spectra for Few-Layered Graphene.

Micro-Raman analysis was performed using a Horiba Jobin-Yvon iHR550 spectrometer with laser excitation at 785 nm, and a wavenumber range of 90 to 3000 cm^{-1}.

The order and disorder of the FLG crystalline structure were studied using micro-Raman spectroscopy. As shown in Figure S5, the Raman spectra display three main peaks: D, G, and 2D. The D peak corresponds to structural C–C vibrational modes, and its intensity can be used to assess the degree of structural disorder. This band is associated with the loss of hybridization in carbon atom [1]. The G peak corresponds to the in-plane stretching modes of sp^2-hybridized carbon, resulting from a double degeneracy in the bond (E_{zg}), and represents the $C(sp)^2 - C(sp)^2$ bond [2–4]. The 2D peak, also known as the G' peak, originates

from a second-order Raman scattering process at the Dirac point [1]. The FLG shows an asymmetry in the 2D peak, which is related to the number of layers in the graphene structure [2].

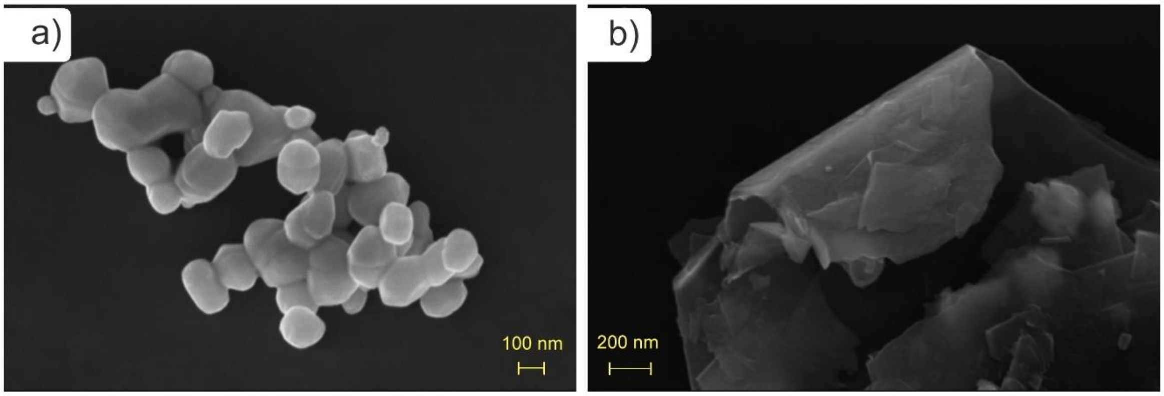

Figure S3. SEM images with magnification of (a) 125000 k × for TiO_2-rutile, and (b) 100000 k × for FLG.

SEM images of TiO_2-rutile and FLG suspensions were obtained using a Zeiss microscope model Supra 35 at 5 kV.

Figure S6 (a) shows SEM images for TiO_2 in the rutile phase. The particles present a tendency to agglomerate. The average size of 119.0 ± 39.5 nm, measured from the SEM images, was obtained using ImageJ. SEM image for FLG is shown in Figure S6 (b), and as observed, the material is organized in a few layers, ranging from 6 to 10 layers.

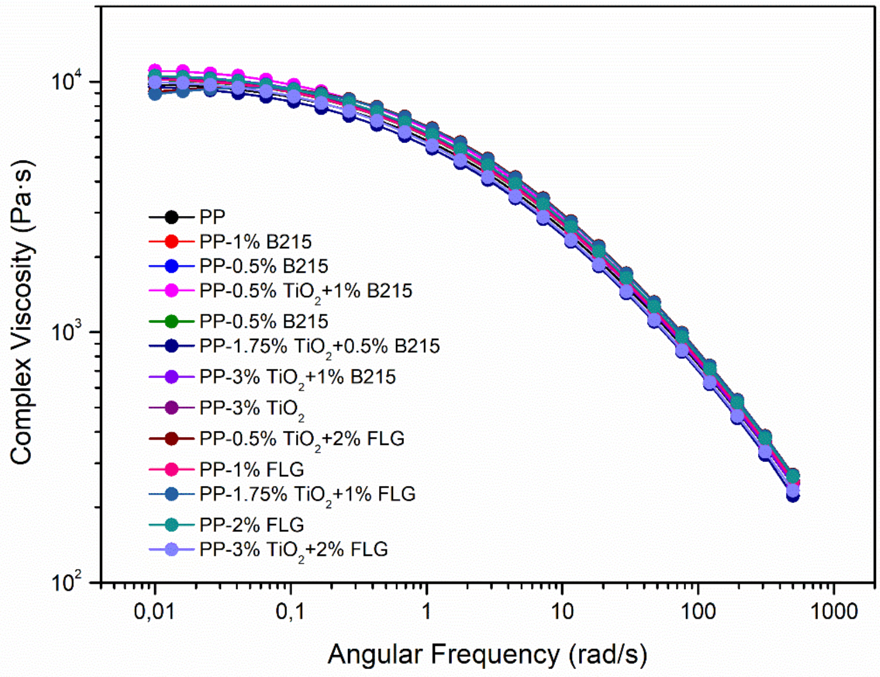

Figure S3. Complex viscosity for all samples without UV-C exposure.

Figure S3 presents the complex viscosity of all samples before UV-C exposure. As shown, the addition of nanostabilizers to the PP matrix resulted in a maximum deviation of ±10% compared to neat PP. The presence of nanostabilizers in the polymer matrix can alter the complex viscosity, either increasing, maintaining, or decreasing its value, depending on the interactions (or lack thereof) between the polymer chains and the nanostabilizers[5]. The low changes in polymer viscosity suggest that the nanostabilizers had a low impact on the complex viscosity of the studied system.

Table S1. Percentage of reduction from the complex viscosity curves for each composite and combination

Sample	η^* (Pa•s)	Reduction %
**PP**	9910982.31	-
**PP 96h**	73461.00	100
**PP 1% FLG 96h**	1730128.17	83

**PP 2% FLG 96h**	4576096.91	54
**PP 0.5% TiO_2 96h**	617157.14	94
**PP 0.5% TiO_2+2% FLG 96h**	2361628.44	76
**PP 1.75% TiO_2+1% FLG 96h**	1912086.45	81
**PP 3% TiO_2 96h**	2653087.06	74
**PP 3% TiO_2+2% FLG 96h**	3525562.21	65
**PP 0.5% B215 96h**	266210.69	98
**PP 1% B215 96h**	382786.91	97
**PP 0.5% TiO_2**	617157.14	94
**PP 0.5% TiO_2+1% B215 96h**	567652.68	95
**PP 1.75% TiO_2+0.5% B215 96h**	1701600.48	83
**PP 3% TiO_2+1% B215 96h**	3610157.53	64

ANOVA

The value for F-Calculated and F – Lack of Fit were obtained from Equations 1 to 6.

- **F-Calculated**

$$F_{\text{regression}} = \frac{\text{Mean Square}_{\text{Regression}}}{\text{Mean Square}_{\text{Error}}} \quad \textbf{(1)}$$

Where:

$$\text{Mean Square}_{\text{Regression}} = \frac{\text{Sum of Squares}_{\text{Regression}}}{\text{Degrees of Freedom}_{\text{Regression}}} \quad \textbf{(2)}$$

And

$$\text{Mean Square}_{\text{Error}} = \frac{\text{Sum of Squares}_{\text{Error}}}{\text{Degrees of Freedom}_{\text{Error}}} \quad \textbf{(3)}$$

- **F – Lack of Fit**

$$F_{\text{Lack of Fit}} = \frac{\text{Mean Square}_{\text{Lack of Fit}}}{\text{Mean Square}_{\text{Pure error}}} \quad \textbf{(4)}$$

Where:

$$\text{Mean Square}_{\text{Lack of Fit}} = \frac{\text{Sum of Squares}_{\text{Lack of Fit}}}{\text{Degrees of Freedom}_{\text{Lack of Fit}}} \quad \textbf{(5)}$$

and

$$\text{Mean Square}_{\text{Pure error}} = \frac{\text{Sum of Squares}_{\text{Pure error}}}{\text{Degrees of Freedom}_{\text{Pure error}}} \quad \textbf{(6)}$$

Table S2. ANOVA for the Viscosity index response for TiO_2 + B215.

Source of Variation	Sum of Squares	Degrees of Freedom	Mean Square	F-value
**Regression**	2.44×10^{-01}	5	4.88×10^{-02}	19.72
**Residual (Error)**	1.98×10^{-02}	8	2.48×10^{-03}	0
**Total**	2.64×10^{-01}	13	2.03×10^{-02}	0
**Lack of Fit**	1.89×10^{-02}	6	3.14×10^{-03}	0.15
**Pure Error**	9.45×10^{-04}	2	4.72×10^{-04}	0
**R^2**	0.92	0.96	-	-
**R^2 max**	0.93	0.96	-	-

Table S3. ANOVA for the Methyl index response for TiO_2 + B215

Source of Variation	Sum of Squares	Degrees of Freedom	Mean Square	F-value
**Regression**	8.70	5	1.74	5.98
**Residual (Error)**	2.33	8	2.91×10^{-01}	0
**Total**	$1.10 \times 10^{+01}$	13	8.48×10^{-01}	0
**Lack of Fit**	1.02	6	1.70×10^{-01}	3.86
**Pure Error**	1.31	2	6.54×10^{-01}	0
**R^2**	0.79	0.89	-	-
**R^2 max**	0.91	0.95	-	-

Table S4. ANOVA for the Global statistical model for TiO_2 + B215

Source of Variation	Sum of Squares	Degrees of Freedom	Mean Square	F-value
**Regression**	1.24	5	2.48×10^{-01}	13.12
**Residual (Error)**	1.51×10^{-01}	8	1.89×10^{-02}	0
**Total**	1.39	13	1.07×10^{-01}	0
**Lack of Fit**	1.10×10^{-01}	6	1.83×10^{-02}	1.13
**Pure Error**	4.14×10^{-02}	2	2.07×10^{-02}	0
**R^2**	0.89	0.94	-	-
**R^2 max**	0.92	0.96	-	-

Table S5. ANOVA for the Viscosity index response for TiO_2 + FLG

Source of Variation	Sum of Squares	Degrees of Freedom	Mean Square	F-value
**Regression**	1.92×10^{-01}	5	3.83×10^{-02}	6.50
**Residual (Error)**	4.72×10^{-02}	8	5.90×10^{-03}	0
**Total**	2.39×10^{-01}	13	1.84×10^{-02}	0
**Lack of Fit**	2.52×10^{-02}	6	4.21×10^{-03}	2.61
**Pure Error**	2.19×10^{-02}	2	1.10×10^{-02}	0
**R^2**	0.80	0.90	-	-
**R^2 max**	0.89	0.95	-	-

Table S6. ANOVA for the Methyl index response for TiO_2 + FLG

Source of Variation	Sum of Squares	Degrees of Freedom	Mean Square	F-value
**Regression**	3.53	5	7.06×10^{-01}	3.21
**Residual (Error)**	1.76	8	2.20×10^{-01}	0
**Total**	5.29	13	4.07×10^{-01}	0
**Lack of Fit**	8.35×10^{-01}	6.	1.39×10^{-01}	3.32
**Pure Error**	9.23×10^{-01}	2	4.62×10^{-01}	0
**R^2**	0.67	0.82	-	-
**R^2 max**	0.84	0.92	-	-

Table S7. ANOVA for the Global statistical model for TiO_2 + FLG

Source of Variation	Sum of Squares	Degrees of Freedom	Mean Square	F-value
**Regression**	9.80×10^{-01}	5	1.96×10^{-01}	7.91
**Residual (Error)**	1.98×10^{-01}	8	2.48×10^{-02}	0
**Total**	1.18	13	9.06×10^{-02}	0
**Lack of Fit**	1.06×10^{-01}	6	1.76×10^{-02}	2.63
**Pure Error**	9.25×10^{-02}	2	4.63×10^{-02}	0
**R^2**	0.83	0.91	-	-
**R^2 max**	0.91	0.95	-	-

Table S8. ANOVA for each combination of photostabilizer, showing the F calculated and the F critical.

ANOVA	F Calculated (Regression)	F Critical (5,8) α=0.05	F Critical (5,8) α=0.01	Regression Significant?	p-value (Regression)	F Calculated (Lack of Fit)	F Critical (6,2) α=0.05	F Critical (6,2) α=0.01	Lack of Fit Significant?	p-value (Lack of Fit)
Global statistical model for TiO_2 + FLG	7.91	3.69	6.63	Yes (5% and 1%)	0.006	2.63	19.33	99.33	No	0.301
Methyl index response for TiO_2 + FLG	3.21	3.69	6.63	No (5%)	0.070	3.32	19.33	99.33	No	0.250
Viscosity index response for TiO_2 + FLG	6.50	3.69	6.63	Yes (5%), No (1%)	0.011	2.61	19.33	99.33	No	0.303
Global statistical model for TiO_2 + B215	13.12	3.69	6.63	Yes (5% and 1%)	0.001	1.13	19.33	99.33	No	0.540
Methyl index response	5.98	3.69	6.63	Yes (5%), No (1%)	0.014	3.86	19.33	99.33	No	0.220

ANOVA	F Calculated (Regression)	F Critical (5,8) α=0.05	F Critical (5,8) α=0.01	Regression Significant?	p-value (Regression)	F Calculated (Lack of Fit)	F Critical (6,2) α=0.05	F Critical (6,2) α=0.01	Lack of Fit Significant?	p-value (Lack of Fit)
for TiO_2 + B215										
Viscosity index response for TiO_2 + B215	19.72	3.69	6.63	Yes (5% and 1%)	<0.001	0.15	19.33	99.33	No	0.867

All ANOVAs show a non-significant Lack of Fit, meaning the models fit the data well. The Global statistical model for TiO_2 + FLG and for TiO_2 + B215, the viscosity index response for TiO_2 + FLG and for TiO_2 + B215, as well as the methyl index response for TiO_2 + B215 have significant regression at 5%, with the Global statistical model for TiO_2 + B215 and the Viscosity index response for TiO_2 + B215 also significant at 1%.

REFERENCES

(1) Mbayachi, V. B.; Ndayiragije, E.; Sammani, T.; Taj, S.; Mbuta, E. R.; Khan, A. ullah. Graphene Synthesis, Characterization and Its Applications: A Review. *Results Chem.* **2021**, *3*, 100163. https://doi.org/10.1016/j.rechem.2021.100163.

(2) Ferrari, A. C.; Meyer, J. C.; Scardaci, V.; Casiraghi, C.; Lazzeri, M.; Mauri, F.; Piscanec, S.; Jiang, D.; Novoselov, K. S.; Roth, S.; Geim, A. K. Raman Spectrum of Graphene and Graphene Layers. *Phys. Rev. Lett.* **2006**, *97* (18), 1–4. https://doi.org/10.1103/PhysRevLett.97.187401.

(3) Gong, J. R. *Graphene – Synthesis, Characterization, Properties and Applications*; Gong, J. R., Ed.; InTech, 2011.

(4) Malard, L. M.; Pimenta, M. A.; Dresselhaus, G.; Dresselhaus, M. S. Raman Spectroscopy in Graphene. *Phys. Rep.* **2009**, *473* (5–6), 51–87. https://doi.org/10.1016/j.physrep.2009.02.003.

(5) Helal, E.; Pottier, C.; David, E.; Fréchette, M.; Demarquette, N. R. Polyethylene/Thermoplastic Elastomer/Zinc Oxide Nanocomposites for High Voltage Insulation Applications: Dielectric, Mechanical and Rheological Behavior. *Eur. Polym. J.* **2018**, *100* (January), 258–269. https://doi.org/10.1016/j.eurpolymj.2018.02.004.
